# *Tet*(C) Gene Transfer between *Chlamydia suis* Strains Occurs by Homologous Recombination after Co-infection: Implications for Spread of Tetracycline-Resistance among *Chlamydiaceae*

**DOI:** 10.3389/fmicb.2017.00156

**Published:** 2017-02-07

**Authors:** Hanna Marti, Hoyon Kim, Sandeep J. Joseph, Stacey Dojiri, Timothy D. Read, Deborah Dean

**Affiliations:** ^1^Center for Immunobiology and Vaccine Development, University of California at San Francisco/Benioff Children’s Hospital Oakland Research Institute, OaklandCA, USA; ^2^Division of Infectious Diseases, Department of Medicine, Emory University School of Medicine, AtlantaGA, USA; ^3^Department of Human Genetics, Emory University School of Medicine, AtlantaGA, USA; ^4^Joint Graduate Program in Bioengineering, University of California, San Francisco, San FranciscoCA, USA; ^5^Joint Graduate Program in Bioengineering, University of California, Berkeley, BerkeleyCA, USA; ^6^Departments of Medicine and Pediatrics, University of California, San Francisco, San FranciscoCA, USA

**Keywords:** *Chlamydia*, tetracycline resistance, transposon, homologous recombination, genomic island, *Chlamydia suis*, *Chlamydia trachomatis*

## Abstract

*Chlamydia suis* is a swine pathogen that has also recently been found to cause zoonotic infections of the human eye, pharynx, and gastrointestinal tract. Many strains contain a tetracycline class C gene [*tet*(C)] cassette that confers tetracycline resistance. The cassette was likely originally acquired by horizontal gene transfer from a Gram-negative donor after the introduction of tetracycline into animal feed in the 1950s. Various research groups have described the capacity for different *Chlamydia* species to exchange DNA by homologous recombination. Since over 90% of *C. suis* strains are tetracycline resistant, they represent a potential source for antibiotic-resistance spread within and between *Chlamydiaceae* species. Here, we examined the genetics of *tet*(C)-transfer among *C. suis* strains. Tetracycline-sensitive *C. suis* strain S45 was simultaneously or sequentially co-infected with tetracycline-resistant *C. suis* strains in McCoy cells. Potential recombinants were clonally purified by a harvest assay derived from the classic plaque assay. *C. suis* strain Rogers132, lacking transposases IS*200* and IS*605*, was the most efficient donor, producing two unique recombinants detected in three of the 56 (5.4%) clones screened. Recombinants were found to have a minimal inhibitory concentration (MIC) of 8-16 μg/mL for tetracycline. Resistance remained stable over 10 passages as long as recombinants were initially grown in tetracycline at twice the MIC of S45 (0.032 μg/mL). Genomic analysis revealed that *tet*(C) had integrated into the S45 genome by homologous recombination at two unique sites depending on the recombinant: a 55 kb exchange between *nrq*F and *pck*G, and a 175 kb exchange between *kds*A and *cys*Q. Neither site was associated with inverted repeats or motifs associated with recombination hotspots. Our findings show that cassette transfer into S45 has low frequency, does not require IS*200*/IS*605* transposases, is stable if initially grown in tetracycline, and results in multiple genomic configurations. We provide a model for stable cassette transfer to better understand the capability for cassette acquisition by *Chlamydiaceae* species that infect humans, a matter of public health importance.

## Introduction

Bacteria develop resistance to antibiotics either as a result of mutation in their chromosomal genes or from acquisition of antibiotic resistance genes by horizontal gene transfer (HGT). Reports of bacterial resistance to antimicrobial agents have occurred almost simultaneously with their first introduction in the late 1930s (Davies and Davies, 2010). Resistance through mutation or HGT is promoted by sub-inhibitory concentrations, broad-spectrum and high doses of antibiotics; patient non-compliance with treatment regimens; and antibiotic use in mammalian and avian species bred for human consumption (Andersson and Hughes, 2014). These latter practices have led to an alarming increase in microbial pathogen resistance such as colistin-resistant *Escherichia coli* and multidrug-resistant *Staphylococcus aureus*. Both have been isolated from pigs and zoonotically transmitted to human hosts (Oppliger et al., 2012; Liu et al., 2016), adding to the crisis in public health infectious disease control (Capita and Alonso-Calleja, 2013; Blair et al., 2015).

Of the five families of obligate intracellular bacteria, including *Ehrlichiaceae. Anaplasmataceae. Rickettsiaceae. Coxiellaceae* and *Chlamydiaceae*, only *Chlamydia suis* has been reported to naturally display antibiotic resistance by acquisition of a resistance gene: a tetracycline resistance class C gene [*tet*(C)]-containing cassette that was acquired by HGT (Dugan et al., 2004; Biswas et al., 2008). *C. suis* is a pig pathogen that causes conjunctivitis, pneumonia, diarrhea/enteritis and reproductive disorders (Schautteet and Vanrompay, 2011; Hoffmann et al., 2015). It has also recently been associated with zoonoses including trachoma (a chronic ocular disease) (Dean et al., 2013), ocular infection in abattoir workers (De Puysseleyr et al., 2014) and asymptomatic nasal, pharyngeal, and intestinal infections in farmers (De Puysseleyr et al., 2015).

Tetracyclines, including doxycycline, are used to treat a variety of bacteria including all *Chlamydia* spp. and, in particular, complicated infections caused by the human pathogen *Chlamydia trachomatis* (Kohlhoff and Hammerschlag, 2015). Since both *C. suis* and *C. trachomatis* infect the human conjunctiva and rectum, the *in vivo* opportunity for HGT of the cassette to *C. trachomatis* is a real concern. Indeed, *C. suis* and *C. trachomatis* co-infections have already been reported among trachoma patients (Dean et al., 2013).

Tetracycline resistance in *C. suis* is conferred by a variable *tet*(C)-containing cassette that encodes an efflux pump to export tetracycline from infected cells. The cassettes are comprised of two or three segments (Dugan et al., 2004; Joseph et al., 2016). One segment, present in all strains, has *tet*(C) and the tetracycline repressor gene *tet*R(C). Another contains replication genes *rep*AC as well as mobilization genes *mob*ABCDE, also present in all strains. A third has two insertion sequences IS*605* and IS*200* that contain transposases. Dugan et al. (2007) showed that these transposases were active in an *Escherichia coli*-based assay. In another study (Suchland et al., 2009), a co-infection model was used to successfully generate tetracycline resistant (tet^R^) *C. trachomatis* L_2_ strains from co-infection of tet^R^ *C. suis* R19 with a tetracycline sensitive (tet^S^) L_2_ strain. However, cassettes lacking the transposases have not been examined for their recombinogenic potential. Here, we tested the requirement of IS*200*/IS*605* transposases for *tet*(C)-containing cassette transfer and developed a model to study cassette transfer among chlamydiae in the presence and absence of tetracycline.

## Materials and Methods

### *Chlamydia* Strains, Cell Culture, and Tetracycline Susceptibility

**Table 1** describes the strains used in this study. All strains were individually propagated in McCoy cells prior to density gradient purification as we previously described (Read et al., 2013; Joseph et al., 2016). McCoy cells were screened for *Mycoplasma* contamination before use (Universal Mycoplasma Detection Kit, ATCC^®^ 30-1012K^TM^, Manassas, VA, USA).

**Table 1:** Characteristics of *Chlamydia suis* strains used in this study.

Strains	Site/Disease	Location	Isolation date	MIC (μg/mL)	Cassette class	Reference
S45	Feces	Austria	1960s	0.016	None	Kaltenboeck et al., 1993^a^
Rogers132	Intestine, lung, conjunctiva	Nebraska, USA	1996	8	II	Dugan et al., 2004^a^
R19	Enteritis	Nebraska, USA	1992	16	I	Dugan et al., 2004^a^
R27	Enteritis	Nebraska, USA	1993	8	IV	Dugan et al., 2004^a^


*^a^Samples obtained from Dr. Art Andersen’s collection (maintained and curated in Dr. Deborah Dean’s lab).*

The *in vitro* tetracycline susceptibility was determined as the minimal inhibitory concentration (MIC) according to Suchland et al. (2003) with minor changes. Briefly, each chlamydial strain was inoculated onto 20 wells of a 48-well plate (E & K Scientific, Santa Clara, CA, USA) seeded with McCoy cells at a multiplicity of infection (MOI) of 0.5 or 1 depending on the infectivity of the strain. After inoculation of cells, the plate was centrifuged at 1500 RPM (Sorvall LegendXTR) for 1 h at 37°C. A tetracycline (Sigma-Aldrich, St. Louis, MO, USA) stock solution (10 mg/mL) in ddH_2_O was used for a twofold dilution in propagation medium consisting of 450 mL Minimal Essential Medium alpha (MEMa, Life Technologies, Carlsbad, CA, USA), 10% Fetal Bovine Serum (FBS, JR Scientific, Woodland, CA, USA), 15 ml sodium bicarbonate (2.8%) (Thermo Fisher Scientific, Waltham, MA, USA), 10 ml glucose (45%) (Fisher Scientific), 10 mM HEPES (Life Technologies) and 1.4 μg/mL Cycloheximide (Sigma-Aldrich) with final concentrations ranging of 0.002 to 256 μg/mL (18 concentrations). After centrifugation, the chlamydial inocula were aspirated and replaced with the serial tetracycline dilutions. Two infected and two uninfected wells received media without tetracycline and served as positive and negative controls, respectively. Cells were fixed with methanol (-20°C) for 10 min after 24–36 h of incubation, depending on the developmental cycle of the strain, at 37°C in 5% CO2. Chlamydial inclusions were detected by direct immunofluorescence using Chlamydia Confirmation Pathfinder (Bio-Rad, Hercules, CA, USA). The MICs were evaluated by analyzing size and morphology as well as the number of inclusions (200 X, Nikon Eclipse Microscope and SPOT imaging software; Diagnostic Instruments, Inc., Sterling Heights, MI, USA). We determined the MIC transition point (MIC_TP_) to be the tetracycline concentrations where 90% or more of the inclusions displayed alterations in size and morphology. Furthermore, the actual MIC was set at twofold higher concentrations (two times the MIC_TP_) as defined by Suchland et al. (2003).

### Generation and Clonal Isolation of Recombinants

**Supplementary Figure S1** shows the schematic and timeline for generation of recombinants. Two different recombination protocols were applied for co-infections. For Protocol 1 (recipient-first before co-infection with donor), confluent monolayers grown in shell vials were inoculated with tet^S^ S45 at an MOI of 4, centrifuged and incubated for 24 h before the addition of tet^R^ parental strains Rogers132, R19, or R27 at an MOI of 0.5. Tetracycline challenge was at 2 μg/mL (1/2 MIC_TP_ Rogers132) as described below. For protocol 2 (simultaneous co-infection), tet^S^ S45 was first grown in McCoy cell monolayers in shell vials to reach 100% infection; 25–50 μL of the infected culture were transferred to a new shell vial with a 80–100% confluent McCoy cell monolayer and simultaneously or immediately consecutively co-infected with tet^R^ R19, tet^R^ R27, or tet^R^ Rogers132. Inoculation was followed by centrifugation for 1 h at 1500 RPM. Tetracycline challenge was with 0.25 μg/mL (8x MIC S45) as described below (**Supplementary Figure S1**).

For each co-infection, three conditions and two controls (single infection with each parental strain) were used. Condition A did not contain any tetracycline (no tet); Condition B contained sub-inhibitory concentrations of tetracycline (1/2 MIC_TP_ for S45; 0.004 (μg/mL); and Condition C contained two times the MIC of S45 (0.032 μg/mL). Co-infected cultures were propagated for 72 h, sonicated once (20% amplitude, Sonic Dismembrator Ultrasonic Processor, Fisher Scientific), and new shell vials were infected to produce 100% infection. Each condition was either directly challenged with tetracycline (2 or 0.25 μg/mL depending on Protocol 1 or 2 described above) or passaged once in propagation medium without antibiotics prior to the tetracycline challenge. Following the challenge for 36–72 h depending on the developmental cycle of the strain, infected cultures were sonicated, and the inoculum was used to perform a harvest assay derived from the classic plaque assay, PCR and sequencing of PCR products to identify putative recombinants (see below, **Supplementary Figure S1**).

Isolation of clonally pure putative recombinants was accomplished using a modified cell culture harvest assay protocol (harvest assay), which derives from the classic plaque assay and is closely related to the shotgun cell culture harvest assay described by Somboonna et al. (2008). Briefly, the first well of a 6-well plate with 60% confluent McCoy monolayers was inoculated with the desired culture; seven serial 10-fold dilutions were performed, of which dilutions 2–7 were applied to wells 1–6. After 24 h, 2 mL of agarose gel (0.5% agarose, Lonza, Rockland, ME, USA) in phenol-red free MEM (Gibco), 10% FBS and 1 μg/ml Cycloheximide was added and topped with propagation medium. After incubation for 4–16 h, the well with detectable but low-level infection was chosen to select individual inclusions (no neighboring inclusions) at 200x magnification. Individual inclusions were picked with a sterile transfer pipet (Fisher Scientific) by punching a hole of 1–2 mm in diameter through the agarose; the plug was then sonicated in propagation medium and used to inoculate one shell vial per picked inclusion containing 500 μL propagation medium. The clones were propagated until the infection reached 100% (3–5 passages). Material from these clones was sonicated and frozen at -80°C, and a paired vial was collected for PCR and sequencing (Somboonna et al., 2011).

### Identification of Putative Recombinants for Genome Sequencing

DNA from collected clones was extracted using the Roche High Pure PCR Product Purification Kit (Roche, Pleasanton, CA, USA), and PCR was performed as previously described (Somboonna et al., 2008). All primers are listed in **Supplementary Table S1**. Clones were considered putative recombinants if they had the following characteristics by PCR: positive for the correct size band for the *tet*(C) gene; positive for the intergenic region (IGR) between the polymorphic membrane protein gene (*pmp*)B and *pmp*C using primers specific for S45; negative for the *pmp*C region using primers specific for Rogers132; and positive for the major outer membrane protein A gene (*omp*A) with confirmation of the S45 *ompA* genotype by Sanger sequencing.

Putative clonal recombinants were then propagated in 0.063 μg/mL tetracycline (4x MIC of S45) to grow stocks for whole-genome sequencing, MIC determination, and the *tet*(C) stability assay (see below). After the first passage, the harvest assay was performed a second time to ensure clonal purity. Picked clones were either grown as described above or directly picked and inoculated into 100 μL HBSS (Gibco) prior to DNA extraction, PCR, and ompA sequencing.

### Genome Sequencing

Stocks of clonally purified putative recombinants were treated with DNase prior to gDNA purification as described previously (Somboonna et al., 2011). Libraries for sequencing were prepared from 0.5 to 1 μg of genomic DNA. Illumina MiSeq libraries were constructed using the Nextera kit and sequenced using the 250 bp paired end protocol on a MiSeq instrument. The resulting sequence data was assembled *de novo* using the SPAdes (Bankevich et al., 2012) software. The CONTIGuator web service (Galardini et al., 2011) was used to map assembled contigs against the Rogers132 strain. We visualized the structure of the assemblies aligned against individual regions of the chromosome using the Bandage graph visualization (Wick et al., 2015) software and its integrated BLAST tool. Querying of short sequences against the raw sequence reads in FASTQ format was performed using the R ShortRead (Morgan et al., 2009) package.

The raw genome data generated for this study are deposited in the SRA database: accession no. SRP096281.

### Analysis of Homologous Recombination and *tet*(C)-Containing Cassette Insertion into *C. suis* Strain S45

We used the parsnp rapid genome alignment tool, which is part of the Harvest suite (Treangen et al., 2014) to identify regions of genome exchange in the recombinants. *De novo* assembled contigs of the recombinants were mapped against the S45 recipient genome reference and compared to the pattern of SNPs obtained when the Rogers132 donor was mapped against S45. Mosaic regions introduced by homologous recombination events appeared as clusters of ‘Rogers132-like’ SNPs in the background of the S45 genome. The approximate boundaries of recombination events were mapped as the edges of continuous runs of inserted SNPs.

### ‘SNP Painting’ to Find Regions of Homologous Recombination in Mixed Cultures

We noted from preliminary analysis that recombinant DNA preparations in some cases also contained residual DNA from donor and/or recipient strains. To visualize the proportion of reads containing the donor and recipient backbone, we used a technique we called ‘SNP painting.’ We extracted two 20mer DNA sequences centered on each SNP identified between the donor and recipient reference sequences that had in their 11th position either the donor or recipient base. We challenged this reference SNP library against a 20mer database created from the FASTQ of the post-mating mixture using Jellyfish software (Marçais and Kingsford, 2011). From the counts of the donor and recipient 20mers at each base, we were able to (1) map recombinant boundaries and (2) ascertain the extent of mixed populations using R software (R Development Core Team, 2015).

### Phylogenetic Analysis

Genes were aligned using PRANK (Löytynoja, 2014). The R package Phangorn (Schliep, 2010) was used to calculate maximum likelihood phylogeny using the Symmetric+GI model (chosen based on best fit). Trees were bootstrapped 100 times.

### MIC Determination (Tetracycline Susceptibility *In vitro*) and *tet*(C) Stability Assay

The MIC of all confirmed recombinants (by PCR and *ompA* genotyping) was tested as described above. To further test the stability of the recombinants regarding the presence of *tet*(C), recombinants were grown at an MOI of 5. Cultures were passaged five times before they were challenged or not with tetracycline using three different conditions: (A) no tetracycline; (B) ½ MIC_TP_ of the recombinant; and (C) 4x MIC of the recombinant. Following challenge, we propagated individual recombinants once under each condition and determined the MIC. Condition A was grown for another five passages (10 total) and was treated similarly as for passage five. The MIC was determined again after one additional passage. Additionally, DNA was extracted from the samples of the recombinants obtained after every passage to confirm that the *omp*A genotype was identical to the recombinant prior to the start of the assay and to test for the presence of *tet*(C) by PCR (**Table 2**).

**Table 2:** List of potential recombinants and their antibiotic susceptibility profiles.

Name	Parental strains	Protocol 1 or 2	Tetracycline condition A, B, or C	*omp*A genotype/132 *pmpC* PCR/S45 *pmpB/C* IGR PCR	*tet*(C) PCR	MIC (μg/mL)
Rec 2	S45/132^a^	(1) 24 hpi, 2 μg/mL	(A) no tetracycline	S45/-/S45	Positive	0.125
Rec 3^b^	S45/132	(1) 24 hpi, 2 μg/mL	(B) ½ MIC_Tp_ S45	S45/-/S45	Positive	8
Rec 4^b^	S45/132	(1) 24 hpi, 2 μg/mL	(B) ½ MIC_Tp_ S45	S45/-/S45	Positive	8
Rec 5^b^	S45/132	(2) Co-inf., 0.25 μg/mL	(B) ½ MIC_Tp_ S45	132/-/S45	Positive	4
Rec 6	S45/132	(2) Co-inf., 0.25 μg/mL	(C) 2x MIC S45	132-S45/132/-	Positive	8
Rec 7	S45/132	(2) Co-inf., 0.25 μg/mL	(C) 2x MIC S45	S45/-/S45	Positive	4
Rec 8	S45/132	(2) Co-inf., 0.25 μg/mL	(C) 2x MIC S45	S45/-/S45	Negative	0.064


*^a^Rogers 132; ^b^true recombinant; (-), negative PCR.*

## Results

### Co-infection of S45 with Tetracycline Resistant Strains R19 and R27 Does Not Yield Recombinants

**FIGURE 1:**
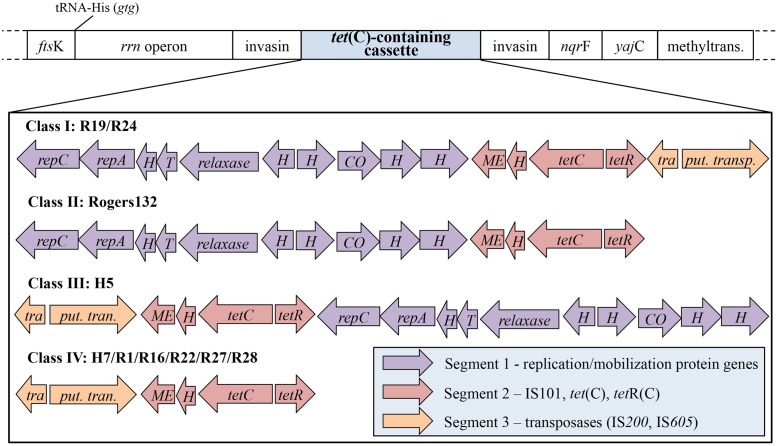
**The structure of the *tet*(C)-containing cassette is comprised of three unique segments.** Shown is the *tet*(C)-containing cassette and neighboring genes, depending on the Class of cassette. The structure of the cassette comprises three diversely arranged segments (purple, red, and orange) consisting of different genes. Four cassette types, termed Class I to IV, have been identified in 11 *C. suis* strains: (I) contains all three segments in the listed segment order (R19 and R24); (II) the cassette of Rogers130 and Rogers132 is lacking segment 3, which contains transposases IS200 and IS605; (III) the cassette of H5 is similar to that of cassette I but occurs in reverse segment order; (IV) is missing segment 1 (replication/mobilization protein genes). *fts*K, DNA translocase gene; *rrn* operon, consisting of 16S, 23S, and 5S rRNA; *nqr*F, Na(+)-translocating NADH-quinone reductase subunit F gene; *yaj*C, preprotein translocase subunit gene; methyltrans., putative RNA methyltransferase gene; *rep*C, replication protein C gene; *rep*A, replication protein A gene; H, hypothetical protein gene; T, Toxin *maz*F gene; relaxase, conjugal transfer relaxase gene; CO, CO dehydrogenase maturation factor gene; ME, mobile element IS101; *tet*C, tetracycline resistant gene class C; *tet*R, tetracycline repressor gene; tetracycline resistance protein class A from transposon 1721; *tra.*, transposase (IS200); *put. trans.*, putative transposase (IS605).

Previous studies have shown that co-infection of the tet^R^ *C. suis* strain R19 with a tet^S^ *C. trachomatis* L_2_ strain results in tet^R^ *C. trachomatis* recombinants (Suchland et al., 2009; Jeffrey et al., 2013). We aimed to obtain *tet*(C)-positive *C. suis* S45 recombinants by co-infecting S45 with three tet^R^ *C. suis* strains representing three of the four *tet*(C)-containing cassette Classes: I (strain R19; complete cassette with all three segments); II (strain Rogers132; without Segment 3); and IV (strain R27; without Segment 1) (**Figure 1**). Eight co-infections with R19 were performed using various protocols, including infection with R19 at 8 or 24 h pi with S45, and pre-treatment of R19 with high doses of tetracycline prior to co-infection. These co-infections tested negative for S45 by PCR using primers specific for the IGR of *pmpB/C* and by *ompA* genotyping (**Supplementary Table S1**) after tetracycline challenge, and were not further investigated. S45 was further grown in shell vials prior to co-infection with R19 (MOI 0.5) at 9 h pi and challenged with 0.25 μg/mL tetracycline. These infections were mixed; however, the 21 inclusions picked via harvest assay either resulted in cultures only positive for R19 by PCR and *omp*A genotyping or in cultures positive for only R19 after two passages (data not shown). One R27 co-infection with S45 was performed and yielded no recombinants.

### Using Sub-inhibitory Tetracycline Concentrations, the *tet*(C) Cassette was Transferred from Rogers132 to S45 Following Co-infection

**FIGURE 2:**
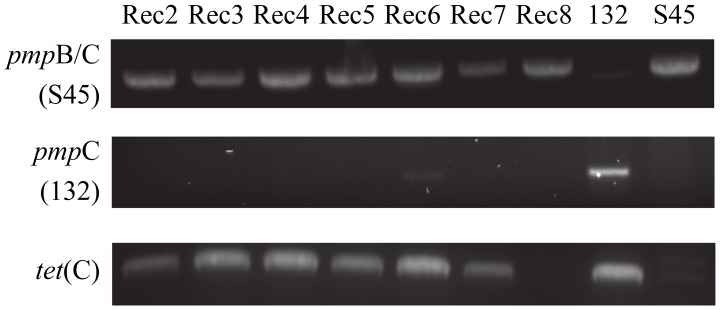
**Strain-specific PCR primers identify putative recombinants.** Shown are the PCR results of each putative recombinant (Rec). Rec 6, a mixed population of S45 and Rogers132, displays a faint positive band for Rogers132-specific *pmp*C (middle lane). All putative recombinants were PCR positive for the S45-specific IGR between *pmp*B and *pmp*C (top lane) and for *tet*[C], bottom row.

Rogers132 (donor strain) and S45 (recipient strain) recombinants were successfully generated by simultaneous or sequential co-infection (see Materials and Methods). The success rate of producing recombinants was low. We obtained seven putative recombinants (Rec 2–8), which were propagated for whole-genome sequencing following a second harvest assay (**Table 2**). In genome analyses described below, several ‘recombinants’ turned out to be mixtures of strains or only parental strains, but the original term is used to describe Rec 2–8. In detail, for co-infection 1, 21 clones were Rogers132; 3 were S45 survivors or mixed infections (including Rec 2); and 2 were true recombinants (Rec 3 and 4). For co-infection 2, 11 were Rogers132; 18 were S45 or mixed infection (including Rec 6–8); and one was a true recombinant (Rec 5). Rec 2 and 8 were tetracycline sensitive after propagation while Rec 6 was positive for both S45 and Rogers132 by *omp*A genotyping and for Rogers132 by *pmp*C PCR. **Figure 2** shows the PCR results for *tet*(C), *pmp*C and *pmp*B/C-IGR for the seven recombinants (**Supplementary Figure S2**: original gel images). We only obtained putative recombinants from co-infections performed in media containing sub-inhibitory (1/2 MIC_TP_) or inhibitory (4x MIC) concentrations of tetracycline for S45; no recombinants were obtained without tetracycline. Only Rec 3, 4 and 5, all originally grown in sub-inhibitory tetracycline concentrations, were later confirmed as true recombinants by genomic analyses (see below).

We evaluated whether confirmed recombinants Rec 3, 4, and 5 remained stable over 10 passages without sub-inhibitory concentrations of tetracycline. After five passages, recombinants were either left drug-free (A) or challenged with low (B) and high (C) doses of tetracycline with subsequent MIC analyses (see Materials and Methods). For each recombinant, every passage was collected and tested for the presence of *tet*(C). At passage 10, *omp*A genotyping was performed to exclude the possibility of a small population of Rogers132 survivors. As expected, Rec 3, 4, and 5 remained positive for *tet*(C) with the identical *omp*A genotype throughout the stability assay (data not shown). These recombinants continued to be tetracycline resistant after passages 5 and 10 with relatively high MICs (**Table 3**; 8–16 μg/mL).

**Table 3:** Stability Assay for five putative recombinants, showing results for passage 5 (P5) and passage 10 (P10).

Name	(*tet*[C] PCR) start to P5 (A)	(*tet*[C] PCR) P5 (B/C)	(*tet*[C] PCR) P6 to P10	MIC (μg/mL) P5	MIC (μg/mL) P10	*omp*A
Rec 3	+/+/+/+/+	+/+	+/+/+/+/+	8	16	S45
Rec 4	+/+/+/+/+	+/+	+/+/+/+/+	8	16	S45
Rec 5	+/+/+/+/+	+/+	+/+/+/+/+	16	16	132^a^
Rec 6	+/+/+/+/+	+/+	+/+/+/+/+	16	16	132^a^
Rec 7	+/+/+/+/+	+/-	-/-/-/-/-	0.25	ND	S45


*^a^Denotes Rogers 132. ND, not done.*

In contrast, putative recombinant Rec 7 was *tet*(C) positive by PCR in the first five passages but negative in passages 6–10 with an S45 *omp*A sequence, while Rec 6 was *tet*(C) positive throughout the assay and tetracycline resistant (**Table 3**).

All recombinants required propagation in 0.064 μg/mL tetracycline (4x MIC S45) in order to produce *tet*(C)-positive chlamydial stocks in sucrose phosphate glutamate (SPG) as described (see Materials and Methods). Attempts to propagate recombinants in the absence of tetracycline led to the loss of the *tet*(C) cassette following serial passages. However, once SPG stocks were produced, the confirmed recombinants were consistently positive for *tet*(C) throughout the 10 passages in the absence of tetracycline for the stability assay, and were phenotypically resistant *in vitro* (MIC > 4 μg/ml).

One co-infection experiment was performed for each of the two protocols under three different tetracycline Conditions: A–C (see **Table 2**). For protocol 1, co-infections were used to infect one 6-well plate per tetracycline Condition. Six to eleven clones were picked (26 total) per plate, resulting in two confirmed recombinants, Rec 3 and 4, from Condition B. For protocol 2, 30 clones were picked with 10 clones each per Condition, resulting in one confirmed recombinant, Rec 5, from Condition B. Taken together, only seven (12.5%) of 56 clones were identified as putative recombinants, and three were confirmed (5.4%). For Condition B, the success rate was 15.0% (3/20).

Altogether, we identified a number of Rogers132 clones (21/26; 80.8%), few S45 survivors and/or mixed infection (3/26 including Rec 2; 11.5%) and two confirmed recombinants (Rec 3 and 4; 7.7%) using Protocol 1 (**Supplementary Figure S1**). This can be explained by the lack of counter-selection used in this study as we wished to observe the outcome of natural co-infection with tetracycline as the only selection method. In contrast, the second co-infection using Protocol 2 (**Supplementary Figure S1**) yielded more S45 survivors or mixed infection (18/30 including Rec 6–8; 60%), several Rogers132 clones (11/30; 36.7%) and only one true recombinant (Rec 5; 3.3%), which can be explained by the abundance of S45 growth in shell vials used for this co-infection experiment as well as the lower dose of tetracycline applied to eliminate S45 survivors (0.25 μg/ml).

### Heterogeneity of *tet*(C) Cassette Insertion into *C. suis* Strain S45

**FIGURE 3:**
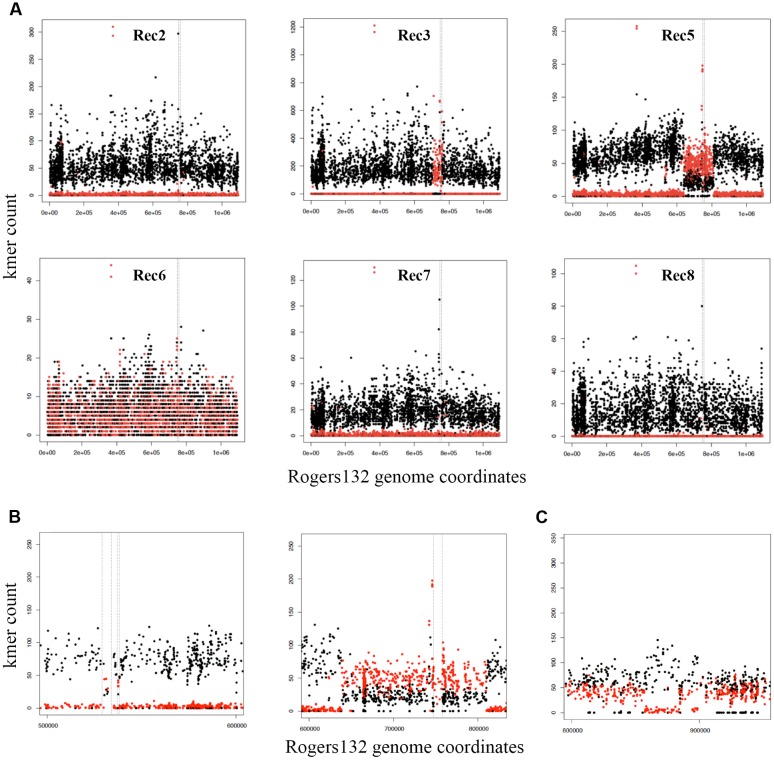
**Identification of Rogers132 insertion(s) and mixed infections in putative recombinant genomes with ‘SNP painting.’** Plots recognizing recombinant regions were created by distinguishing the two parental strains Rogers132 (red) and S45 (black) (see Materials and Methods). **(A)** Schematic of the putative recombinants Rec 2, 3, 5–8 clockwise from top left exemplifying crossover regions in Rec 3 and 5; **(B)** Enlarged region of the two recombinant regions found in Rec 5, and **(C)** Schematic representation of recombinant regions within Rec 6, a putative recombinant, which was a mixed infection. The SNP painting plot of the Rec 6 co-infection shows the region that maps to Rogers132 between coordinates 800000 to 950000. The region between ∼856,780–884,340 has low to zero coverage of Rogers132 type SNPs (red) and higher coverage of the S45, suggestive of recombination within the region. The Rogers132 strain donor has acquired DNA from the S45 recipient.

The seven putative recombinants described above were genome sequenced (**Supplementary Table S2**). We made a database of 4,864 SNPs between Rogers132 and S45 using parsnp (Treangen et al., 2014) (**Datasheet S1**) and used them as markers to distinguish potential recombination junctions in raw sequence data (**Figure 3**, ‘SNP painting’: see Materials and Methods). Aside from Rec 6, which was a mixture of donor and recipient, the recombinants were predominantly comprised of the S45 genetic backbone, evincing our success in screening for the recipient following co-infection. Recombinant regions were determined at the approximate junctions between regions of ‘S45-like’ and ‘Rogers-like’ SNPs (**Figure 3A**). While Rec 2 was PCR-positive for *tet*(C), there was undetectable recombination based on genome sequence analysis. Similarly, Rec 7 had no evidence for recombination. Rec 3 and 4 were confirmed recombinants with identical *tet*(C) cassette insertion sites at Rogers132 coordinates of ∼705,300–760,600 (upstream and downstream cross-overs varied in length because of sparse SNPs) in the S45 backbone, constituting ∼55,300 nucleotides (5.3% of the genome) including the *rrn* operons of 16S, 23S, and 5S rRNAs. The insertion spans from the Na(+)-translocating NADH-quinone reductase subunit F gene (*nqr*F) to the phosphoenolpyruvate carboxykinase [GTP] gene (*pck*G).

Rec 5 had a much larger insertion containing the *tet*(C) cassette at Rogers132 coordinates ∼638,000–813,000 (**Figures 3A,B**, right plot), spanning the 2-dehydro-3-deoxyphosphooctonate aldolase gene (*kds*A) to the 3′(2′), 5′-bisphosphate nucleotidase gene (*cys*Q) in the S45 backbone. This region constitutes 175,000 nucleotides (16.6% of the genome). There were also second and third insertions within Rogers132 coordinates 529,000–538,000 in the S45 backbone (**Figure 3B**, left plot), including the putative general secretion pathway protein D gene (*gsp*D) to the secretion system effector C gene (*sse*C) family. The presence of a subpopulation of S45-like SNPs across the 638,000–813,000 recombinant region revealed that Rec 5 was a mixed culture, consisting of a majority recombinant population and a minority of S45 recipient containing no Rogers132 DNA.

Although Rec 6 was a mixture of donor and recipient (**Figure 3A**), we identified a recombinant region of ∼40,000 nucleotides between Rogers132 coordinates ∼856,780–884,340 (**Figure 3C**). These coordinates are in the *pmp*D and phenylalanine t-RNA ligase (*phe*S) genes (**Figure 3C**). Rec 8 had no evidence of recombinants, consistent with the PCR results (**Figure 2**), and was considered an S45 survivor.

**FIGURE 4:**
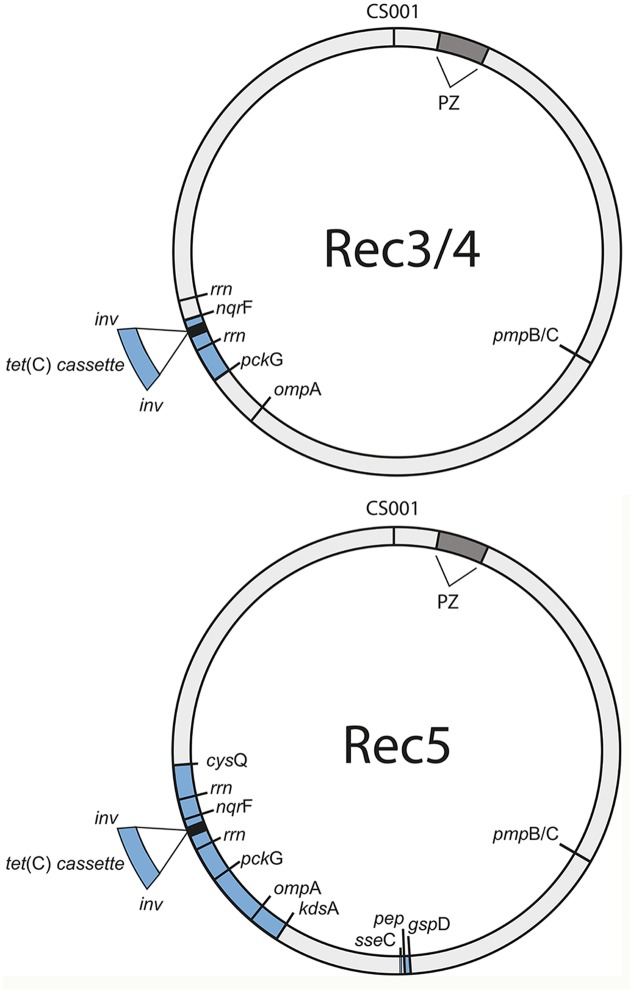
***Tet*(C)-cassette insertions in the S45 genome are heterogeneous.** Shown are the sites of insertion (blue) for the three confirmed recombinants, Rec 3/4 (top) and Rec 5 (bottom), in the circular S45 genome. PZ, plasticity zone (dark gray); *cys*Q, adenosine-3′(2′),5′-bisphosphate nucleotidase; rrn, rrn operon (16S rRNA, 23S rRNA, 5S rRNA); *nqr*F, Na(+)-translocating NADH-quinone reductase subunit F; *pck*G, phosphoenolpyruvate carboxykinase [GTP]; *omp*A, major outer membrane protein A; *kds*A, 2-dehydro-3-deoxyphosphooctonate aldolase; *gsp*D, Putative general secretion pathway protein D; hp, hypothetical protein.

**FIGURE 5:**
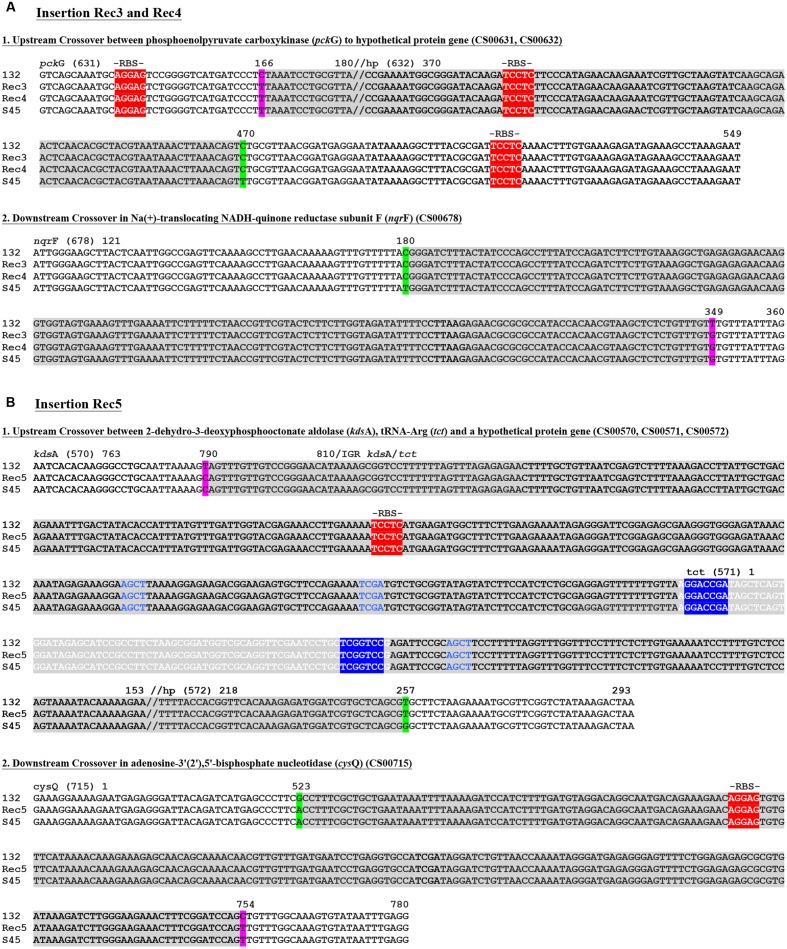
**Recombinant crossover regions in Rec 3 and 4, and Rec 5 based on sequence alignments with S45 and Rogers132.** Shown are the nucleotide sequences of the crossovers up- and down-stream for Rec 3 and 4 **(A)** and the large insertion of Rec 5 **(B)**. Numbers on top of the sequences represent the positions relative to the respective gene. Gene IDs (e.g., CS00631) refer to the annotation of Rogers132. Crossover regions are highlighted in gray with SNPs indicated in magenta if the recombinants aligned to the S45 backbone and in green if they aligned to Rogers132. Palindromes are indicated by blue letters, while tRNAs are highlighted in light gray with white letters. The tRNA associated inverted repeats are highlighted in blue with white letters. Putative ribosomal binding sites (RBS) are highlighted in red and further indicated by “-RBS-” on top of the nucleotide sequence.

**Figure 4** shows the recombinant locations of the cassette and other insertion sites within the context of the entire circular genome for Rec 3 and 4, and for Rec 5. While the cassette was confirmed to reside within *ilp* and *rrn* operons for all 11 previously genome sequenced *C. suis* strains (Joseph et al., 2016), the insertion in Rec 3 and 4 included *nqr*F, extending to *pck*G beyond the downstream *rrn* operon. The upstream crossover is located within ∼2.2 kb, either in *pck*G or its neighboring gene downstream, encoding a hypothetical protein, and in proximity to ribosomal bindings sites (RBSs), whereas the downstream crossover is found within *nqr*F (**Figure 5A**).

**FIGURE 6:**
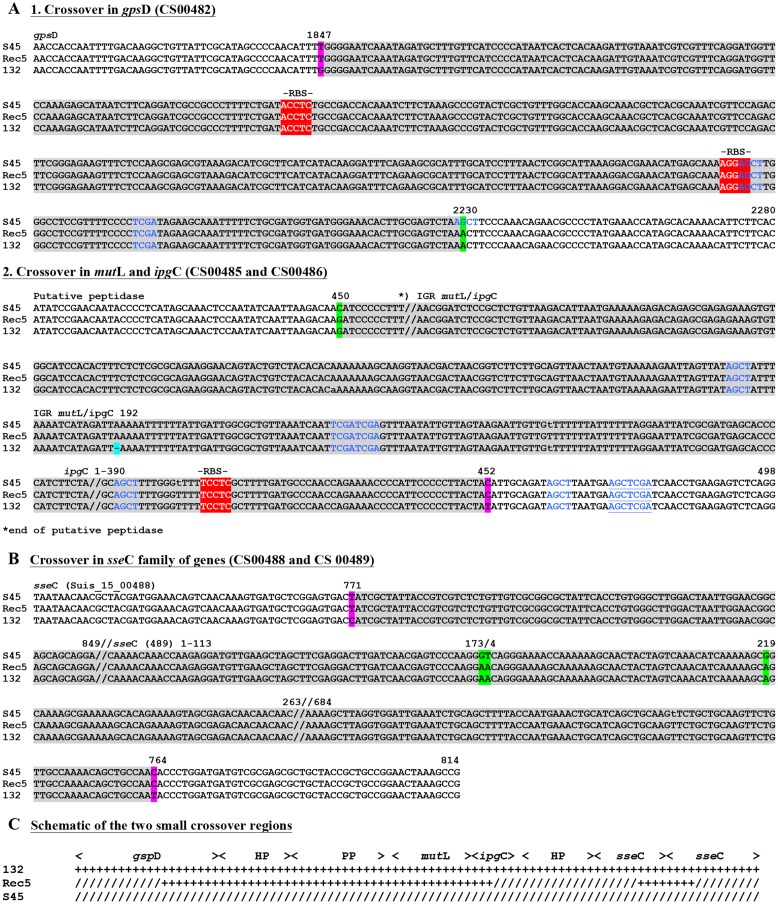
**Recombinant crossover regions for the two small insertion for Rec 5 based on sequence alignments with S45 and Rogers132.** Shown are the nucleotide sequences of the small insertion of Rec 5. **(A)** Represents the slightly longer region upstream, while **(B)** shows the shorter crossover region downstream. Numbers on top of the sequences represent the positions relative to the respective gene. Gene IDs (e.g., CS00631) refer to the annotation of Rogers132. Crossover regions are highlighted in gray with SNPs indicated in magenta if the recombinants aligned to the S45 backbone and in green if they aligned to Rogers132. Blue letters indicate palindromes while putative RBS are highlighted in red and further indicated by “-RBS-” on top of the nucleotide sequence. **(C)** Shown is the schematic overview of the two small crossover regions.

For Rec 5, the cassette insertion spanned *cys*Q to *kds*A, which included the *rrn* operons and the *omp*A gene of Rogers132. The upstream crossover is located within ∼750 bp between the downstream end of *kds*A or tRNA-Arg (*tct*) and a hypothetical protein-encoding gene, while the downstream crossover is found in *cys*Q, spanning 230 bp (**Figures 4** and **5B**). Both crossovers are in proximity to RBSs, and palindromes are evident. We also identified two shorter recombinant regions located adjacent to each other between *kds*A and *pmp*BC (**Figure 4**). The larger of the two crossovers was found upstream within *gps*D (∼380 bp) and downstream, either in *mut*L or *ipg*C, spanning ∼3 kb (**Figure 6A**), and in proximity to RBSs with evidence for palindromes where one is at the site of the SNP at nucleotide position 2230 (**Figure 6A1**). The complete crossover located within the *sse*C-like gene family spanned 1500 bp (**Figure 6B**). **Figure 6C** shows a schematic of the two Rogers132 insertions.

In examining the plasmids, all except Rec 7 had the S45 plasmid. Rec 6 was a mixture of both S45 and Rogers132 plasmids. None of the plasmids exhibited any genetic exchange.

## Discussion

Mutation, HGT, and genome rearrangement shape bacterial genomes on an evolutionary time scale (Darmon and Leach, 2014). Most *Chlamydia* genomes, of which *C. trachomatis* is the best studied species, show evidence of intra-species recombination events (Millman et al., 2001; Gomes et al., 2007; Jeffrey et al., 2010; Mitchell et al., 2010; Joseph et al., 2011, 2012, 2015, 2016; Harris et al., 2012; Joseph and Read, 2012; Putman et al., 2013; Read et al., 2013; Bachmann et al., 2015) but little to no evidence for classic HGT acquired pathogenicity islands (Nunes and Gomes, 2014) or other foreign genes except for bacteriophage inserts in *Chlamydia pneumoniae* (Rosenwald et al., 2014) and genes possibly acquired in the plasticity zone (Read et al., 2000; Liu et al., 2007). The most notable exception to this rule is *C. suis*, which is not only known for the possession of the *tet*(C)-containing cassette (Dugan et al., 2004), but has recently been shown to be highly recombinogenic compared to other *Chlamydia* species (Joseph et al., 2016).

To date, four classes of *tet*(C)-containing cassettes have been described (Donati et al., 2014; Joseph et al., 2016). The cassettes are inserted at an identical site within the chromosomal invasin gene (called *ilp* or *inv*-like gene) flanked upstream by an *rrn* operon and downstream by the gene *nqr*F (Dugan et al., 2004; Joseph et al., 2016). It is likely that the cassette originated from one ancestral transposition event, although other options are possible (Joseph et al., 2016). Tet^S^ strain S45 possesses the intact *ilp* gene, which is conserved within the *C. suis* species (**Supplementary Figure S3**) (Donati et al., 2016) but also found in *C. caviae* strain GPIC [AE015925.1], sharing 73% identity and 91% query cover by BLASTN (Liu et al., 2007). No other *Chlamydia* species or other known species contains this gene. These data suggest that *ilp* is required for cassette insertion. However, Suchland et al. (2009) demonstrated that *C. muridarum* strain MoPn and *C. trachomatis* strain L_2_, both of which lack *ilp*, are able to acquire the cassette *in vitro* by co-infection with *C. suis* strain R19, while *C. caviae* was not receptive.

In a follow-up study, Jeffrey et al. (2013) co-infected *tet*(C)-positive and -negative L_2_ recombinants from the former study with non-LGV *C. trachomatis* strains F and J to produce new tet^R^ recombinants. Neither study found specific nucleotide sequences that suggested a mechanism for *in vitro* recombination, although the cassette between the *rrn* operons of R19 was inserted into the downstream *rrn* operon of L_2_. In addition to a similar localization of the cassette in the *rrn* operon, Jeffrey et al. (2013) identified what appeared to be non-specific recombination (190 events in 12 recombinant strains) throughout the genome, unlike the R19/L_2_ recombinants, suggesting that the progeny may have contained mixed infections despite selection of clones by limiting dilution.

In contrast to the successfully produced tet^R^ *C. trachomatis* L_2_ and *C. muridarum* recombinants with R19 (Suchland et al., 2009), we were unable to produce any *tet*(C)-positive *C. suis* S45 recombinants after co-infection with R19. Similarly, co-infections with R27 were unsuccessful because R27 outgrew S45 within 2–3 passages. These observations were surprising especially if we consider that resistance-determining accessory resistance genes are thought to generally impair rather than promote biological fitness of bacteria in the absence of antibiotics (Andersson and Levin, 1999). One possible explanation is that S45 was isolated in the 1960s and adapted to cell culture in the laboratory, whereas the tet^R^ strain donors have only been cultured since the 1990s. To test this hypothesis, other Class I and IV *C. suis* strains with a similar cassette would have to be co-infected with a more recently isolated tet^S^ *C. suis* strain.

As opposed to previous co-infection studies, we implemented three different tetracycline conditions and included S45 sub-inhibitory (0.004 μg/mL) and inhibitory (0.032 μg/mL) tetracycline concentrations. Suchland et al. (2009) performed co-infections without antibiotics prior to challenge with high-dose tetracycline. Our conditions were based on the hypothesis that low concentrations of antibiotics promote the selection toward resistant bacteria (Cantón and Morosini, 2011). Indeed, all three confirmed recombinants (Rec 3, 4, and 5) were originally grown in sub-inhibitory concentrations, suggesting that mating is optimized by a multiplicity of donors. Only one putative recombinant was isolated from cultures that were not initially grown in tetracycline, which was later confirmed as an S45 survivor (Rec 2). To confirm that sub-inhibitory concentrations of tetracycline promote the transfer of the *tet*(C) cassette, quantitative analysis would be necessary. For example, replicates of independent co-infection experiments could be performed with 20–30 clones picked per tetracycline condition instead of 6–10 as in our study. The number of recombinants could then be compared among tetracycline conditions.

Dugan et al. (2007) previously proposed that one or both IS*605* transposases were responsible for integration of the cassette into the *C. suis* chromosome. While the initial HGT event that brought the ancestral *tet*(C)-containing cassette into *C. suis* probably involved transposition, we were able to demonstrate that transfer of the cassette between *C. suis* was through double crossover homologous recombination. This suggests that homologous recombination has been a significant factor in the recent spread of tetracycline resistance among *C. suis* strains where 89–100% are resistant in the US, Europe and the Middle East (Dugan et al., 2004; Di Francesco et al., 2008).

The boundaries of our recombinants were not near the duplicated *rrn* operons as in the tet^R^ *C. trachomatis* recombinants and one of 12 sequenced recombinants described by Suchland et al. (2009) and Jeffrey et al. (2013), respectively, but rather in conserved, syntenic genome regions (**Figures 5** and **6**). In our previous study, comparative genomics was used to infer that putative ancestral recombination had occurred at high frequency across the *C. suis* genome (Joseph et al., 2016). The recombination boundaries in Rec 3 and 4 were identical, suggesting they arose from sibling plaques rather than independent events, and overlapped genes in recognized recombinant regions (CS00632 and CS00678) (Joseph et al., 2016), but the large Rec 5 recombination region did not (genes CS00570–572, CS00715). Furthermore, small insertions in Rec 5 were incorporated within one non-recombinant (CS00485) and two recombinant regions (CS00482, CS00488–489). It is unclear, with our limited number of *C. suis* genomes to compare to date, whether the observed patterns reflect selection, recombination hotspots or are purely stochastic. In inspecting the regions, inverted repeats, chi sites or direct target repeats that are typical permissive sites for recombination were not detected. However, the upstream crossover in Rec 5 contained a tRNA (**Figure 5B**). These genes are known to be acquired and involved in recombination for a diversity of bacteria (Bishop et al., 2005; McDonald et al., 2015). Conserved regions such as RBSs, which were present within or near each cross-over region in our study (**Figures 5** and **6**), may also facilitate homologous recombination because gene function would not be altered, allowing new recombinants to be successful (Gomes et al., 2007) as in the present study. A similar lack of patterned recombination was noted in a recent study of beta-lactam and vancomycin resistance in *Enterococcus faecium*, in which the authors hypothesized that long sequences of highly homologous DNA were targets for recombination (García-Solache et al., 2016). Since at least 89% of *C. suis* strains isolated from farm animals are tet^R^ (Andersen and Rogers, 1998; Di Francesco et al., 2008) and *C. suis* shares 79.8% average nucleotide identity with *C. trachomatis*, the potential for homologous recombination and cassette transfer are high.

In summary, we present a co-infection model that produces recombinants, demonstrating for the first time that the *tet*(C)-containing cassette is transferred between *C. suis* strains by homologous recombination without the need for IS*200*/IS*605* transposases (Cassette Segment 3). We also discovered that, while the frequency of recombination is low, sub-inhibitory concentrations of tetracycline may promote transfer and that, rather than targeting highly polymorphic regions, recombination occurs in long homologous sequences and genomic regions with tRNAs. Our model will serve as a template for determining the mechanisms and frequency of cassette transfer among *Chlamydia* species including *C. trachomatis* that may co-infect humans at the same anatomic sites as tet^R^ *C. suis* zoonotic strains. Cassette transfer would have major implications for public health approaches to treatment for humans and domesticated animals alike.

## Author Contributions

Substantial contribution to the conception or design of the work: HM, DD, TR. Acquisition, analysis, interpretation of data: HM, HK, SJ, SD, TR, DD. Draft and/or critical revision of the manuscript: HM, DD, TR, SJ, SD, HK. Final approval of the version to be published: HM, DD, TR, SJ, SD, HK. All authors agree to be accountable for all aspects of the work.

## Conflict of Interest Statement

The authors declare that the research was conducted in the absence of any commercial or financial relationships that could be construed as a potential conflict of interest.

## Supplementary Material

**FIGURE S1 | Co-infection protocols and conditions.**

**FIGURE S2 | Strain-specific PCR primers identify putative recombinants (original gels).**

**FIGURE S3 | Phylogeny of invasin gene of *C. suis* and *C. caviae*.**

**TABLE S1 | Primers used to identify putative recombinants.**

**TABLE S2 | Sequencing statistics for the seven putative recombinants.**

**DATASHEET S1 | Database of 4,864 SNPs identified between the Rogers132 donor and S45 recipient.**
