# Supplementary material for: Tet(C) Gene Transfer between Chlamydia suis Strains Occurs by Homologous Recombination after Co-infection: Implications for Spread of Tetracycline-Resistance among Chlamydiaceae
Source: Front Microbiol. 2017 Feb 7;8:156. doi: 10.3389/fmicb.2017.00156 (PMC5293829; doi:10.3389/fmicb.2017.00156)
Supplement: Supplementary file 1 [file Image_1.PDF]

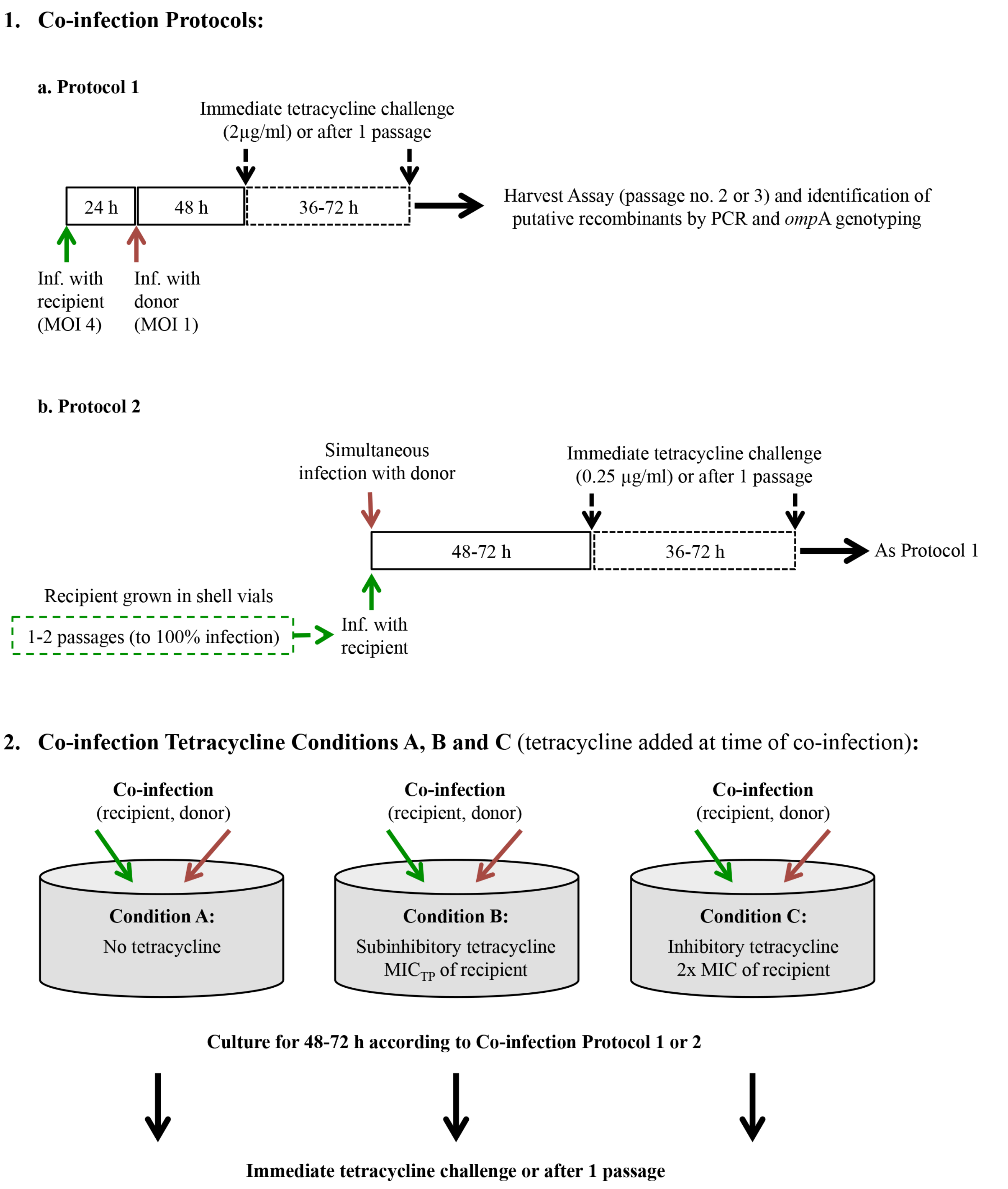

Figure S1. Co-infection Protocols and Conditions. 1) Shown are the two co-infection protocols, which consisted of a) staggered infection of the donor (strains R19, R27 and Rogers132) 24 hours post infection with the recipient strain S45 (Protocol 1) and b) simultaneous co-infection of recipient and donor after the recipient was first grown to 100% infection in shell vials (Protocol 2). 2) Shown are the culturing conditions at the time of co-infection without tetracycline (Condition A), subinhibitory concentrations of tetracycline (Condition B) and inhibitory concentration of tetracycline (Condition C) for the recipient strain.
