# Supplementary figures and images for: Tet(C) Gene Transfer between Chlamydia suis Strains Occurs by Homologous Recombination after Co-infection: Implications for Spread of Tetracycline-Resistance among Chlamydiaceae
Source: Front Microbiol. 2017 Feb 7;8:156. doi: 10.3389/fmicb.2017.00156 (PMC5293829; doi:10.3389/fmicb.2017.00156)

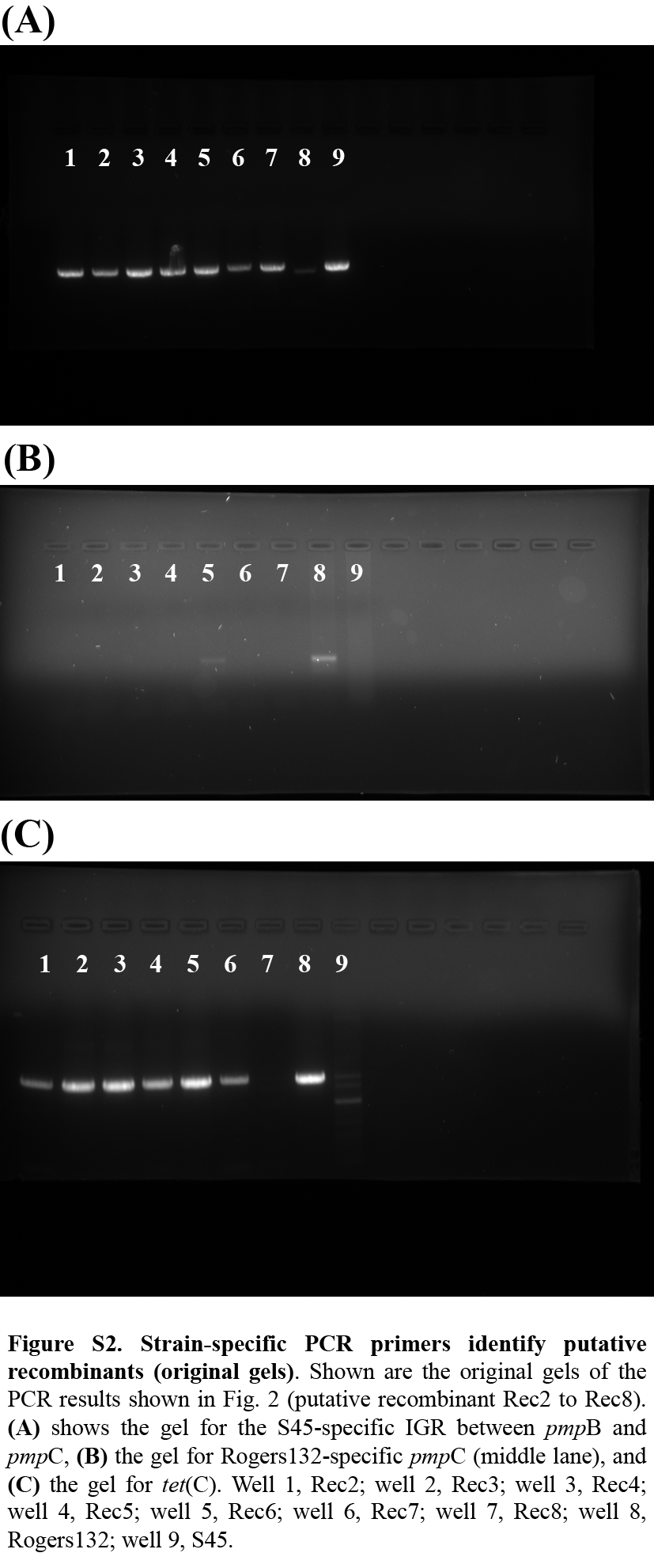

Supplement: Supplementary file 2 [file Image_2.TIF]

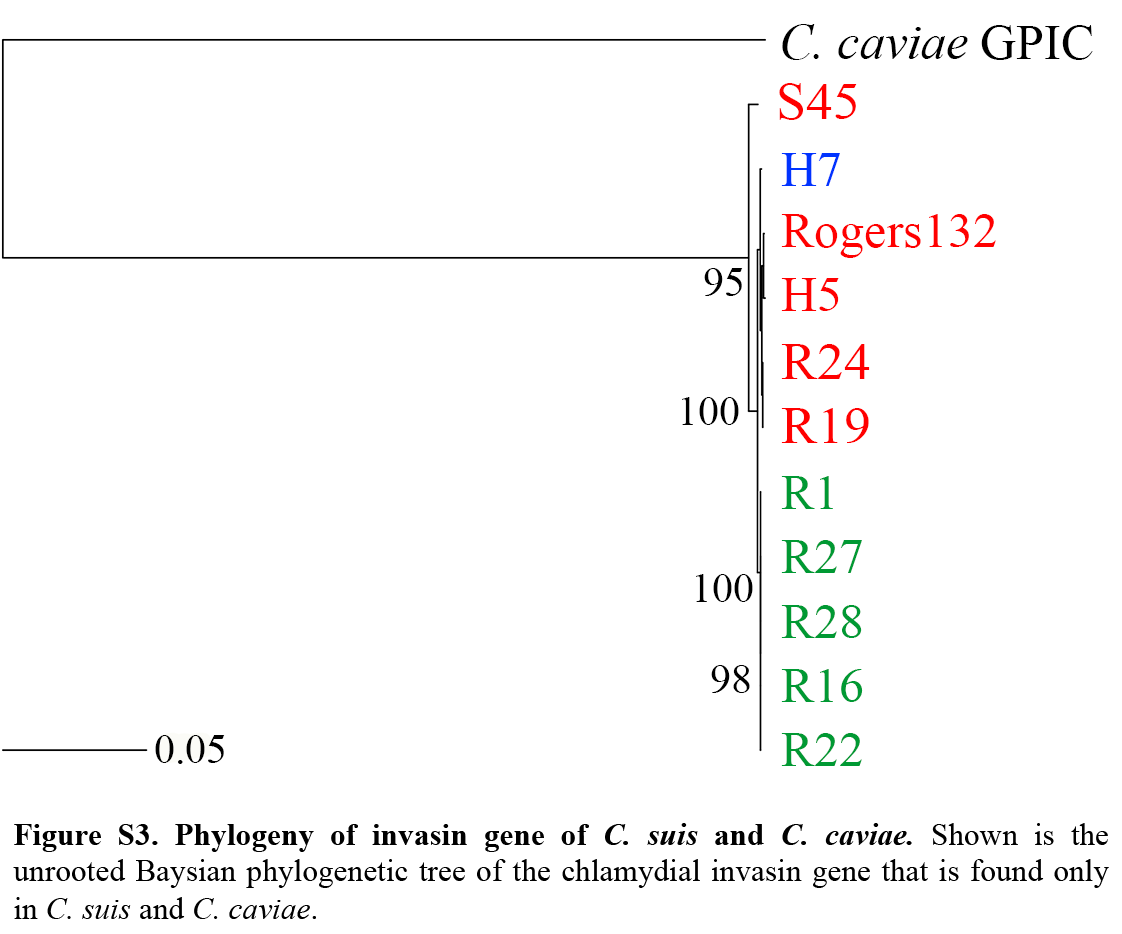

Supplement: Supplementary file 3 [file Image_3.TIF]
