# Supplementary material for: Tet(C) Gene Transfer between Chlamydia suis Strains Occurs by Homologous Recombination after Co-infection: Implications for Spread of Tetracycline-Resistance among Chlamydiaceae
Source: Front Microbiol. 2017 Feb 7;8:156. doi: 10.3389/fmicb.2017.00156 (PMC5293829; doi:10.3389/fmicb.2017.00156)
Supplement: Supplementary file 4 [file Table_1.PDF]

Supplemental Material

Tet(C) gene transfer between *Chlamydia suis* strains occurs by homologous recombination after co-infection: Implications for spread of tetracycline-resistance among *Chlamydiaceae*

Hanna Marti[a*], Hoyon Kim[a], Sandeep J. Joseph[b,c], Stacey Dojiri[a], Timothy D. Read[b,c], Deborah Dean[a,d]#

Center for Immunobiology and Vaccine Development, UCSF Benioff Children's Hospital Oakland Research Institute, Oakland, California, USA[a]; Department of Medicine, Division of Infectious Diseases[b] and Department of Human Genetics, Emory University School of Medicine, Atlanta, Georgia, USA[c]; Joint Graduate Program in Bioengineering, University of California, San Francisco, California, USA, and University of California, Berkeley, Berkeley, California, USA[d].

Table S1

Target information		Primer 1 (5' to 3')			Primer 2 (5' to 3')		
**Location**	**Length (bp)**	**Forward/ Reverse**	**Name**	**Sequence**	**Name**	**Sequence**	**Reference**
tetracycline gene class C [*tet(*C)]	589	-	CS43	AGCACTGTCCGACCGCTTTG	CS47	TCCTCGCCGAAAATGACCC	Dugan et al. 2004
IGR between *pmp)*B and *pmp*C specific for S45	257	Forward	pmp - CsS45 - 1F	GAAGAAAACTCACAAACGTCCT	IGR-S45 - 2F	GAAGAACCTACTCACCCAGAG	this study
		Reverse	pmp - CsS45 - 1R	GGACGTTTGTGAGTTTTCTTCT	IGR-S45 - 2R	CTCTGGGTGAGTAGGTTCTTC	
*pmp*C specific for Rogers132	269	Forward	pmp - Cs132 - 1F	GGAGAGCCCAAATACAGTTACA	pmp132 - 1R	GTAACTGTATTTGGGCTCTCCA	this study
		Reverse	pmp - Cs132 - 2F	GAAGAAACTACTCACCCAGAG	pmp132 - 2R	CTCTGGGTGAGTAGTTTCTTC	
*pmp*C specific for R19	271	Forward	1F	CAGTTACAGAAGAAAACCCAC	pmpR19-1R	TGTGGGTTTTCTTCTGTAACT	this study
		Reverse	2F	CCAGAGTCTACAGAAGAAGAT	pmpR19-1F	TTGATCTTCTTCTGTAGACTCT	
Outer membrane protein gene *(omp*A)	1278	-	ReverseCt*	TAGAATCTGAATTGAGCGTTTACGTGA	ForwardCt	GGACATCTTGTCTGGCTTT	this study

* is the sequencing primer of *omp*A
