# Supplementary material for: Tet(C) Gene Transfer between Chlamydia suis Strains Occurs by Homologous Recombination after Co-infection: Implications for Spread of Tetracycline-Resistance among Chlamydiaceae
Source: Front Microbiol. 2017 Feb 7;8:156. doi: 10.3389/fmicb.2017.00156 (PMC5293829; doi:10.3389/fmicb.2017.00156)
Supplement: Supplementary file 5 [file Table_2.PDF]

**Supplemental Material**

# *Tet*(C) gene transfer between *Chlamydia suis* strains occurs by homologous recombination after co-infection: Implications for spread of tetracycline-resistance among *Chlamydiaceae*

Hanna Marti[a*], Hoyon Kim[a], Sandeep J. Joseph[b,c], Stacey Dojiri[a], Timothy D. Read[b,c], Deborah Dean[a,d]#

Center for Immunobiology and Vaccine Development, UCSF Benioff Children's Hospital Oakland Research Institute, Oakland, California, USA[a]; Department of Medicine, Division of Infectious Diseases[b] and Department of Human Genetics, Emory University School of Medicine, Atlanta, Georgia, USA[c]; Joint Graduate Program in Bioengineering, University of California, San Francisco, California, USA, and University of California, Berkeley, Berkeley, California, USA[d].

**Table S2.** Sequencing statistics for the seven putative recombinants

| Name | Total bp | read_total | read_max | qual_media | qual_25th_percent | qual_75th_percent | Raw Chlamydia coverage equivalent* |
|---|---|---|---|---|---|---|---|
| Rec1 | 108905832 | 474706 | 250 | 35 | 31 | 37 | 103.71984 |
| Rec2 | 1915016067 | 10078184 | 250 | 37 | 34 | 38 | 1823.824826 |
| Rec3 | 206338749 | 946292 | 250 | 36 | 32 | 37 | 196.5130943 |
| Rec4 | 328887114 | 1528798 | 250 | 36 | 34 | 37 | 313.2258229 |
| Rec5 | 139202659 | 676362 | 250 | 37 | 35 | 38 | 132.573961 |
| Rec6 | 1.53E+09 | 8278242 | 250 | 37 | 34 | 38 | 1457.152381 |
| Rec7 | 7.43E+08 | 3618692 | 250 | 36 | 34 | 38 | 707.7190476 |
| Rec8 | 1.33E+09 | 8829746 | 250 | 37 | 35 | 38 | 1270.485714 |

* assumes that 100% of the DNA was Ct.  Actually it was 50% or less

*Chalmydia* genome = 1,050,000 bp
